# Identification of Metabolism-Associated Biomarkers for Early and Precise Diagnosis of Oral Squamous Cell Carcinoma

**DOI:** 10.3390/biom12030400

**Published:** 2022-03-04

**Authors:** Yuhan Wang, Xiaoxin Zhang, Shuai Wang, Zihui Li, Xinyang Hu, Xihu Yang, Yuxian Song, Yue Jing, Qingang Hu, Yanhong Ni

**Affiliations:** 1 Central Laboratory of Stomatology, Nanjing Stomatological Hospital, Medical School of Nanjing University, Nanjing 210008, China; dg20350111@smail.nju.edu.cn (Y.W.); sissi.117@hotmail.com (X.Z.); ws0826@outlook.com (S.W.); mf21350182@smail.nju.edu.cn (Z.L.); mf21350178@smail.nju.edu.cn (X.H.); songyuxian1986@126.com (Y.S.); jingyueshadow@126.com (Y.J.); 2 Department of Oral and Maxillofacial Surgery, Affiliated Hospital of Jiangsu University, Zhenjiang 210008, China; yangxihu1981@126.com; 3 Department of Oral and Maxillofacial Surgery, Nanjing Stomatological Hospital, Medical School of Nanjing University, Nanjing 210008, China

**Keywords:** oral squamous cell carcinoma, metabolic reprogramming, biomarker, early diagnosis, precise diagnosis

## Abstract

The 5-year survival rate for oral squamous cell carcinoma (OSCC), one of the most common head and neck cancers, has not improved in the last 20 years. Poor prognosis of OSCC is the result of failure in early and precise diagnosis. Metabolic reprogramming, including the alteration of the uptake and utilisation of glucose, amino acids and lipids, is an important feature of OSCC and can be used to identify its biomarkers for early and precise diagnosis. In this review, we summarise how recent findings of rewired metabolic networks in OSCC have facilitated early and precise diagnosis of OSCC.

## 1. Introduction

Oral squamous cell carcinoma (OSCC) is the most common head and neck malignancy, and its incidence has been increasing in several countries [1]. Treatment is mainly based on surgery, with radiotherapy and/or chemotherapy as adjuncts. Frustratingly, treatments have not significantly prolonged the survival of such patients [2,3], and the 5-year survival rate has remained at approximately 60% for the last 20 years [4]. The poor prognosis of OSCC patients is partly because of delayed diagnosis. Early screening and timely therapeutic intervention can effectively arrest OSCC progression, thereby increasing patient survival by 80% [5,6]. OSCC diagnosis relies on clinician examination. However, some symptoms of OSCC appear similar to those of oral ulcers or precancerous lesions, leading to confusion. This phenomenon suggests that early screening for OSCC requires extensive experience. The gold standard for definitive diagnosis of OSCC is pathological diagnosis, which is invasive and leads to pain and poor wound healing [7]. In addition to the cumbersome histopathology procedures, sampling at different sites may result in different pathological diagnoses, and the complex procedures of pathological diagnosis cause a certain lag in obtaining clinical results [8,9]. Therefore, in clinical practice, there is an urgent demand for diagnostic tools with high specificity, manipulability and non-invasive or minimally invasive techniques to assist clinicians in OSCC screening. Once patients are suspected of having OSCC, a precise diagnosis that assists surgeons in planning surgery and predicting patient therapy responses is urgently required. For example, the difficulty in determining a ‘clear’ surgical margin is an important factor that influences the prognosis of OSCC. We previously reported that OSCC patients with mild dysplasia margins had a worse prognosis than those with negative margins [10]. Moreover, excess resection seriously affects the quality of life of patients. Hence, only a precise diagnosis can help characterise every patient based on molecular signatures and provide personalised treatments with predictable outcomes.

Metabolic rewiring is one of the six hallmarks of cancer, manifesting mainly as alterations in glucose, lipid and amino acid metabolism. Tumour cells experience complex stresses, including hypoxia, energy deprivation and an acidic environment, and must adapt to environmental pressures through metabolic reprogramming, which can be used to design metabolism-targeted diagnostic tools. For example, positron emission tomography, which records energy utilisation, is recommended for patients with head and neck cancer at clinical stages III and IV, and exhibits diagnostic advantages for detecting lymph node and distant metastasis [11]. Metabolic features vary across tumours of different tissue origins, genetic backgrounds and stages of the disease. Therefore, nuclear magnetic resonance (NMR) spectroscopy, mass spectrometry (MS), ambient ionisation MS and conductive polymer spray ionisation MS (CPSI-MS) have been performed to compare the metabolic variation among OSCC patients with different clinical stages and genetic backgrounds of the disease to identify novel metabolic landscapes of OSCC. Using these findings, early and precise diagnosis of OSCC should soon be realised.

In the last few years, many promising, innovative diagnostic techniques, such as narrow-band imaging, high-frequency ultrasounds, optical coherence tomography and in vivo confocal microscopy, have been applied as adjunctive non-invasive techniques to help diagnose OSCC [12]. Owing to detailed studies on metabolic pathways and tremendous advancements in techniques, the application of metabolite-targeted diagnosis in clinical settings has drawn great attention and shows promise. In this review, we analyse 93 papers and present an overview of how OSCC induces metabolic changes to adapt to a nutrient-poor environment and confer growth advantages to tumour cells. Unlike other reviews that focus on the metabolic characteristics of OSCC [13,14], this review classifies the identified metabolites according to sample types and their diagnostic values and discusses how these metabolites can be potentially applied for early and precise OSCC diagnosis.

## 2. Altered Cellular Metabolism in OSCC

### 2.1. Glucose Metabolism

#### 2.1.1. Glycolysis

**Figure 1:**
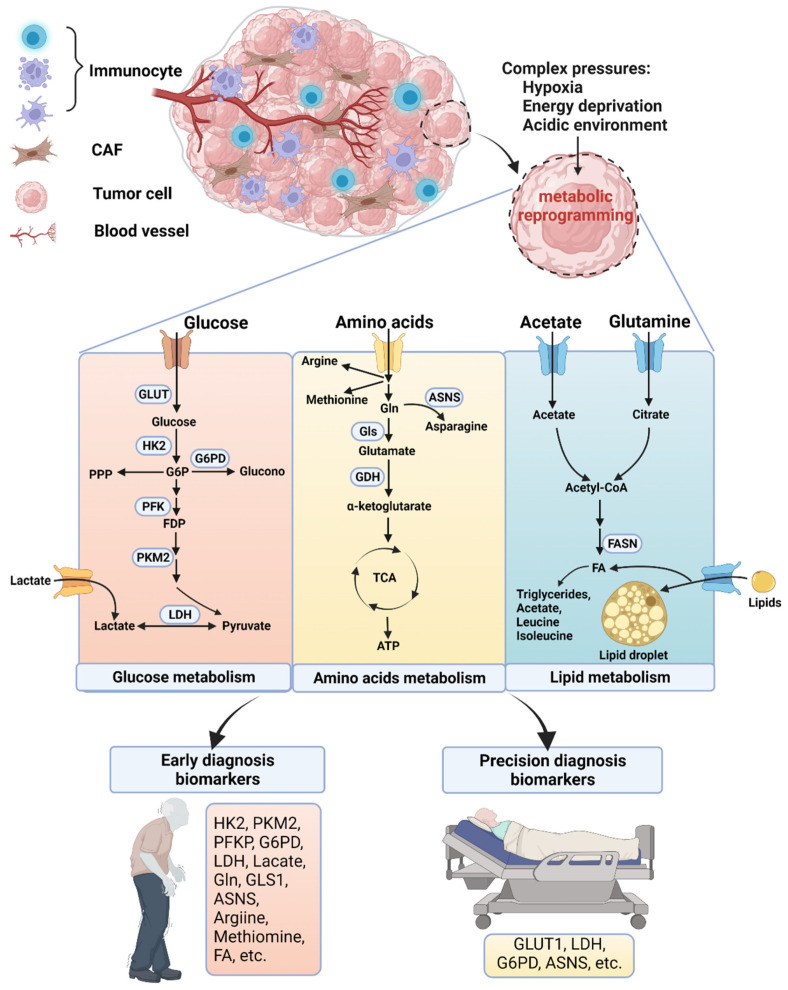
Screening altered cellular metabolism to diagnose OSCC. OSCC undergoes metabolic reprogramming of glucose, lipids and amino acids in response to complex pressures. Identifying metabolism-associated biomarkers facilitates early and precision diagnosis. CAF, cancer-associated fibroblast; LDH, lactate dehydrogenase; Gln, glutamine; ASNS, asparagine synthetase; Gls, glutaminase; GDH, glutamate dehydrogenase; TCA, tricarboxylic acid; ATP, adenosine triphosphate; FA, fatty acid; FASN, fatty acid synthase. Figure created with biorender.com.

One of the earliest findings in cancer metabolic reprogramming was that tumour cells prefer glycolysis even in the presence of adequate oxygen. Glycolysis addiction in OSCC is evidenced by enhanced glucose uptake, which is mainly reflected by the upregulation of glucose transporter protein (GLUT) [15]. Higher GLUT1 and GLUT3 expression correlates with poor prognosis in OSCC patients [16,17,18,19,20,21]. In addition, when glucose is transported into the cytoplasm, it is catalysed by many glycolytic enzymes, among which hexokinase 2 (HK2), pyruvate kinase M2 (PKM2), phosphofructokinase (PFK) and glucose-6-phosphate dehydrogenase (G6PD) have also been shown to be upregulated in OSCC and associated with OSCC patient prognosis [22,23,24,25,26] (Figure 1). These results demonstrate that OSCC is characterised by enhanced glycolysis activity.

Enhanced glycolysis is accompanied by increased lactate production. LDH, which converts pyruvate to lactate, has been monitored in serum and saliva to detect and diagnose OSCC [27,28,29,30] (Figure 1). Analysis of LDH expression in tumour tissues can also be used to predict patient prognosis and chemotherapy responses [31,32,33]. Notably, abundant lactate in OSCC is produced not only by malignant cells but also by other cells in the tumour microenvironment, such as CAF. Lactate in the tumour microenvironment can be employed by tumour cells as a nutrient to promote cell proliferation and invasion [34,35,36,37]. Lactate is also a signalling metabolite, which lactylates histones to regulate gene expression [38]. The high levels and multiple functions of lactate in OSCC suggest that it is a promising glycolytic metabolite for OSCC detection and diagnosis.

#### 2.1.2. Pentose Phosphate Pathway (PPP)

When glucose enters the cytoplasm and is phosphorylated by HK to glucose-6-phosphate, it enters the PPP in addition to glycolysis (Figure 1). PPP contributes to OSCC progression by maintaining intracellular redox homeostasis, FA synthesis, and the production of ribose 5-phosphate for RNA and DNA synthesis [39]. The rate-limiting enzyme of PPP, G6PD, is regulated by NRF2 and associated with poor prognosis of OSCC [25], hence, G6PD-targeting limits cancer growth and metastasis by increasing reactive oxygen species (ROS) levels and endoplasmic reticulum stress [40,41]. Although few studies have further confirmed that PPP is enhanced in OSCC, three independent studies have demonstrated that transketolase (another PPP enzyme) is overexpressed in head and neck squamous cell carcinoma (HNSCC) cell lines and tissues [42,43,44]. Additionally, radiosensitive HNSCC cells display higher PPP activity than radioresistant cells [45]. Therefore, further research is required to confirm enhanced PPP activity in OSCC and to identify the key metabolites.

### 2.2. Amino Acid Metabolism—Gln and Methionine

It is now widely appreciated that tumour cells are characterised by not only dysregulated glucose metabolism but also enhanced requirements for amino acids [46]. Normal cells cannot synthesise essential amino acids (histidine, isoleucine, leucine, lysine, methionine, phenylalanine, threonine, tryptophan and valine) and obtain them exogenously. In contrast, tumour cells have an increased dependence on exogenous non-essential amino acids and display enhanced activity of amino acid synthesis, breakdown, and transport because amino acids can provide energy, regulate redox balance, and support protein and lipid synthesis [47]. 

Gln, the second primary nutrient for tumours, is a non-essential amino acid that is most abundant in circulation. Some tumour cells, including neuroblastoma [48], clear cell renal cell carcinoma [49], and breast cancer [50] cells, are addicted to Gln; hence, Gln depletion undermines their cell proliferation. As tumours consume more Gln than that required for biosynthesis, it must be transported from outside by Gln transporters. Fourteen amino acid transporters are responsible for the influx/efflux of Gln into/out of cells [51]. Among these transporters, ASCT2 (SLC1A5) exhibits higher affinity for Gln and is upregulated in OSCC [52,53]. Luo et al. found that depleting Gln by inhibiting ASCT2 impairs OSCC proliferation and tumour growth; this indicates the importance of Gln in OSCC [53]. 

Gln plays multiple roles in OSCC progression (Figure 1). Firstly, Gln replenishes the TCA cycle via α-ketoglutarate (α-KG) to synthesise citrate and FA. Specifically, Gln is catalysed by Gls to glutamate, which is then converted to α-KG by GDH. Multiple research teams have confirmed that Gls is highly expressed in OSCC and that its expression correlates with poor prognosis in OSCC patients [54,55,56]. Moreover, Gls regulates the radiosensitivity of HNSCC cells [57,58]. Chang et al. found that the use of Gln by p53-regulated Gls confers ROS resistance onto tumour cells [57]. GLUD expression in OSCC has not been observed. Only Cetindis et al. found weak expression of GLUD in OSCC [52], suggesting that Gln in OSCC may not participate in the TCA cycle to generate ATP. Secondly, Gln serves as a nitrogen donor to generate nucleotides and non-essential amino acids. Glutamate can be converted to asparagine by ASNS (Figure 1). Our group found that higher ASNS expression in OSCC positively correlated with lymph node metastasis and perineural invasion [59]. This implies that ASNS has the potential to be a significant factor for predicting the prognosis of OSCC patients. In addition, glutamate is a precursor of glutathione, which exerts antioxidant effects. Moreover, Gln contributes to the import of some essential amino acids. Furthermore, mitochondrial Gln is a precursor of 2-hydroxyglutarate, which increases stem cell marker expression [60]. 

Methionine, a methyl donor, contributes to the initiation and progression of OSCC via epigenetic modifications. Methionine adenosyltransferase generates S-adenosyl methionine using methionine as a substrate. DNA methyltransferases and histone methyltransferases then transfer a methyl group to cytosine or histones, respectively, to activate or repress gene expression. Owing to its importance, C-11-methionine positron emission tomography positron emission tomography (MET-PET) is clinically applied for some tumours to assist in diagnosis. Chowdhury et al. compared fluorodeoxyglucose-PET (FDG-PET) and MET-PET for oral cancer and showed that the uptake values of methionine and glucose are similar. Both uptakes increase in patients at a higher clinical stage [61]. More notably, Saleha et al. found that D-methionine protected normal oral tissue from radiation-induced cell death [62]. Therefore, MET-PET can be further applied to assess patient responses to radio therapy.

In addition to the aforementioned amino acids, OSCC may also rely on other amino acids, such as arginine [63,64]. All these studies confirmed that compared with normal cells, OSCC cells have different amino acid preferences and utilisation rates. Therefore, the levels of amino acids and their metabolising enzymes are promising diagnostic values; these are discussed below.

### 2.3. Lipid Metabolism

Lipid is a general term for various organic compounds, including glycerolipids, glycerophospholipids, sphingolipids and cholesterol [65]. Lipids are an outstanding medium for energy storage [66,67] and are essential components of biological membranes [68,69]. They also transmit signals as vehicles [70], act as activators [71], or enzyme carriers involved in signal recognition, and participate in immunity responses [72]. A wide variety of lipids with diverse functions have constructed a massive, flexible network to fulfil the requirements of malignant cells. 

Multiple studies have shown that the genes related to lipid metabolism are dysregulated in OSCC and that some of them are associated with patient prognosis and clinical features. Hu et al. found that obesity is an independent risk factor for early OSCC and that three genes responsible for lipid metabolism are predictors of prognosis [73]. Similarly, Gao et al. identified a 24-gene set related to lipid metabolism that could be used to predict OSCC prognosis, assist in diagnosis and choose rational treatments [74]. In addition, lipid metabolism-related proteins are differentially expressed in OSCC with variable differentiation [75]. All these results prove that lipid metabolism is dysregulated in OSCC.

FA uptake and synthesis are active in cancer cells. FA uptake is aided by low-density lipoprotein receptor, CD36, FA transporter proteins and FA binding proteins (Figure 1). CD36 in OSCC has been extensively studied and found to correlate with OSCC proliferation, migration and lymph-node metastasis [76,77,78]. Downregulating CD36 expression inhibits OSCC progression [77,79]. In addition, fatty-acid-binding protein 5 promotes OSCC migration [80]. Endogenous FA are synthesised from acetyl-CoA, which is then converted to malonyl-CoA by acetyl-CoA carboxylases. FASN then elongates acetyl-CoA to yield palmitate. FASN is upregulated in OSCC, and higher FASN expression in OSCC is associated with advanced disease and poor prognosis [81,82,83]. FASN not only promotes OSCC proliferation and migration but also enhances cell resistance to chemotherapy [84,85,86]. The de novo synthesised FA further connect via different backbones to form various lipids. For example, phospholipids consist of two FA, a glycerol unit and a phosphate group which is esterified to an organic molecule such as choline, glycerol or inositol. Hilvo et al. showed that de novo synthesised FA are incorporated into membrane phospholipids of breast cancer cells, and hence have diagnostic value [87].

Cholesterol also plays essential roles in tumorigenesis and cancer progression by forming membranes, modulating signals, and contributing to bile acid and steroid hormone synthesis. Cholesterol metabolism is altered in OSCC, and high cholesterol levels promote oral carcinogenesis [88,89]. Shutting down cholesterol efflux by silencing apolipoprotein E expression impairs OSCC invasion [90]. Multiple studies have shown that cholesterol synthesis is important for cancer cells. However, the function of cholesterol synthesis in OSCC remains undiscovered. 

Saliva prostaglandin E_2_ (PGE2) is another potential marker for OSCC diagnosis [91]. Li et al. found that PGE2 promotes OSCC proliferation. In addition to the direct detection of PGE2, cyclooxygenase (COX)-2, which is responsible for PGE2 production, has also been widely studied [92]. COX-2 expression is elevated in OSCC [93]. COX-2 promotes OSCC invasion and proliferation, which is partially dependent on PGE2 [94].

Owing to the close correlation between reprogrammed lipid metabolism and tumour progression, lipid metabolism enzymes and lipodomics have diagnostic potential. Although numerous studies have been conducted to identify markers for tumour diagnosis, no reliable markers have been found, and few are currently applied in clinical settings. The difficulty lies in technology insensitivity and various confounders (including patient fasting status and metabolic medications) [95]. However, owing to great advances in technology and a more detailed stratification of patients, reliable markers will be identified in the future.

## 3. Clinical Applications of Metabolism-Targeted Diagnosis

Since OSCC metabolic reprogramming is recognised and altered metabolites have many effects on OSCC cell phenotypes, OSCC can potentially be screened by detecting changes in metabolites to determine disease malignancy and formulate appropriate treatment plans. The association between metabolites in liquid samples (saliva, serum and urine) of patients with OSCC or premalignant lesions and healthy individuals is one research focus. The correlation between metabolite levels in tumour tissues and clinical characteristics or prognosis has also been extensively studied. Herein, we summarise the recent developments.

### 3.1. Metabolism-Targeted Early Diagnosis

The main reason for delayed diagnosis of OSCC is the difficulty in distinguishing OSCC from other oral premalignant lesions using accurate non-invasive or minimally invasive strategies that are equivalent to histological diagnosis. Therefore, researchers have compared metabolites in easily collectible fluids from patients with OSCC or premalignant lesions and normal individuals to identify typical OSCC metabolites to assist physicians in diagnosis.

#### 3.1.1. Metabolism-Targeted Early Diagnosis—Saliva

Saliva, which is readily available and can be non-invasively obtained, is the ideal choice of sample for OSCC diagnosis. Saliva is a mixture of water (93–94%), organic and inorganic substances (0.2%), proteins, and numerous cellular elements (0.3%) which is produced by salivary glands located throughout the oral mucosa. In addition to the aforementioned molecules, saliva contains gingival crevicular fluid, serum transudate, epithelial cells, leukocytes, and many microorganisms. The various contents of saliva maintain oral homeostasis via lubrication, buffering, taste, digestion, and antibacterial, antiviral, and antifungal protection [96]. Individuals with different physiological and pathological conditions produce different saliva [97]. Therefore, many studies have attempted to compare saliva from patients with OSCC and normal individuals. Recently, capillary electrophoresis time-of-flight MS (CE-TOF-MS), gas chromatography coupled with MS (GC-MS), and ultraperformance liquid chromatography-MS (UPLC-MS) were used to profile metabolites in saliva, and several typical metabolites, including glycolysis metabolites, amino acids and lipids, were identified [98,99,100,101] (Table 1). We further analysed the most differential metabolites among these studies and found that five metabolites—taurine, valine, choline, cadaverine and tryptophan—had been simultaneously identified using three independent detection methods, indicating their application potential [99,100,101]. Notably, all these metabolites were hydrophilic because of the limitations of a single chromatographic method. To overcome this shortcoming, Wang et al. developed an integrated separation approach using reversed-phase liquid chromatography and hydrophilic interaction chromatography combined with TOF-MS, and identified five potential markers (propionylcholine, *N*-acetyl-l-phenylalanine, sphinganine, phytosphingosine and S-carboxymethyl-l-cysteine) [102].

Early diagnosis of OSCC requires rapid equipment feedback. However, metabolic analysis of saliva using these types of equipment is time-consuming. Therefore, our group introduced an ambient-ionisation-based multiplex molecular screening method called CPSI-MS. The analysis time was reduced to a few seconds from the few weeks or months required when using traditional methods [103]. We showed that the diagnostic accuracy could reach 86.7% upon combining CPSI-MS with machine learning.

In addition, studies have been conducted to compare metabolites in tumours and premalignant lesions. Shigeo et al. found that 14 metabolites were significantly different in OSCC and oral lichen planus groups [112], confirming that saliva metabolites in oral leukoplakia and OSCC differed significantly (Table 1). They compared saliva samples using CE-MS and identified a panel of indole-3-acetate and ethanolamine phosphate to discriminate OSCC from oral lichen planus [112]. Similarly, Wei et al. used UPLC and identified a panel of valine, lactic acid and phenylalanine to distinguish OSCC from oral leukoplakia [108]. 

#### 3.1.2. Metabolism-Targeted Early Diagnosis—Serum and Urine

In addition to saliva, serum and urine have been studied using metabolomics for early OSCC diagnosis. Q-TOF-LC-MS, GC-MS and ^1^H NMR have been applied to discriminate OSCC from normal or oral leukoplakia [109,118]. However, we found that the number of patients in some of the aforementioned studies was small, which may have led to poor generalisability and stability of the results. This indicates that increasing the amount of patient data is necessary for validation. Therefore, we further compared the metabolites of serum from healthy individuals and 578 patients with OSCC using CPSI-MS [110]. Sixty-five metabolites were identified as potential markers. The accuracy of distinguishing individuals with OSCC from normal individuals was 98% in the discovery cohort and 89.6% in the validation cohort. This study is the largest metabolic study on serum for early OSCC diagnosis to date.

### 3.2. Metabolism-Targeted Precision Diagnosis

In addition to early diagnosis, a precise diagnosis to guide surgeons in operating and formulating treatment strategies is indispensable for improving OSCC prognosis and quality of life of patients. Some equipment targeting altered metabolism has been applied to precision diagnosis. For example, FDG-PET is recommended for patients with HNSCC. This technique displayed high sensitivity and accuracy for screening distant metastases and altered the management of 13.7% of patients [119,120,121]. In the following subsections, we summarise recent findings in metabolism-targeted precision diagnosis.

#### 3.2.1. Metabolism-Targeted Precision Diagnosis—Body Fluids

Fluids, including saliva, serum and urine, are not only assayed to discriminate patients with OSCC from healthy individuals or individuals with oral premalignant lesions but also have precise diagnostic values. Since some metabolites in fluids reflect reprogrammed tumour metabolism, they are associated with clinical characteristics or histopathological grades. For example, glycolysis-related metabolites (pyruvate and lactate) in serum correlate with patients with OSCC at higher clinical stages or of more advanced histopathological grades [122]. Abnormal metabolic amino acid levels can also be used to determine OSCC prognosis. Notably, 600 MHz NMR has been used to successfully analyse amino acid metabolomics in plasma, and a panel of amino acids to determine lymph node metastasis has been found [115]. Similarly, serum lipid levels, including those of cholesterol, high-density lipoprotein and low-density lipoprotein, have been reported to gradually decrease with the development and progression of OSCC [111]. 

In addition to being indicators of clinical characteristics, metabolites in fluids have also been used to predict recurrence and therapy efficacy. Zuo et al. used UPLC-quadrupole/Orbitrap high-resolution MS to compare OSCC metabolites before and after operation and found that OSCC was less likely to recur in patients with low succinic acid and high hypoxanthine levels [123]. Furthermore, Ye et al. found that the metabolites related to glycolysis, redox homeostasis and anabolic progress could be used to predict chemotherapy efficacy with an accuracy of 100%, 81.25% and 100.0% in the training, test and external validation sets, respectively [124].

#### 3.2.2. Metabolism-Targeted Precision Diagnosis—Tissue Specimens

Instead of measuring metabolites in fluids to indirectly reflect the characteristics of OSCC, many studies have focused on metabolic variations in tumour tissue. Traditional pathological diagnosis has proven that many metabolic-related enzymes are associated with patient prognosis and therapy sensitivity. However, these enzyme-dependent tests are not sufficient to accurately describe the metabolic signatures of OSCC. Therefore, researchers have detected metabolites in tissue specimens. Their findings show that the metabolites in tumours are associated with tumour invasion, neuropathic pain, and lymph-node metastasis [116,119,125]. Metabolic shifts have diagnostic value, similar to metabolite levels. Mignion analysed the relationship between lactate and pyruvate levels by adding isotopic markers to metabolites and created pyruvate–lactate dynamic metabolic images, which correlated with epidermal growth factor receptor inhibitor resistance in HNSCC [126].

In comparison with those in adjacent normal tissue, metabolites change in OSCC [14,113,114,115,116,127]. However, the metabolic trends in normal tissue and tumours were unknown until a study was conducted by Young et al., wherein the metabolic perturbation of distance-related surgical margins was analysed and four and six amino acids were identified as negative margin and dysplastic margin markers, respectively [114]. This work was particularly important as they tried to identify a panel of metabolites at the junction of normal and tumour tissues to determine the safe surgical margin. Using a reliable panel of identified metabolites, rapid evaporative ionisation MS (REIMS), which captures the gaseous ions generated during the cutting of cancer tissue with an electric knife and constructs a metabolomic profile of the corresponding tissue, will be translated from the laboratory bench to clinical application [128]. In addition, acquiring a vivid metabolic image of OSCC is also useful for surgeons, especially during surgery. Uchiyama et al. distinguished the cancer and stromal regions of OSCC using imaging MS [129]. Young et al. further applied DESI-MS imaging and developed 14 lipid ion molecular diagnostic models to measure safe surgical resection distances for OSCC [130]. This attempt was successful in using OSCC lipid metabolomics to guide the surgical treatment of OSCC and identify small tumour foci at the surgical margin. It may be possible to determine the ‘cleanliness’ of surgical margins in real-time by surgical margin metabolite detection in the operating room to eliminate the hidden danger of residual tumour foci. Currently, the assessment of surgical safety margins using DESI-MS for gastric [131], prostate [132] and breast cancers [133] is highly compatible with the pathological results. We believe that the clinical application of REIMS and DESI-MS during surgery has the potential to make individualised surgical safety margins possible.

## 4. Future Research Directions of Metabolomics Applied to OSCC Diagnosis 

**Figure 2:**
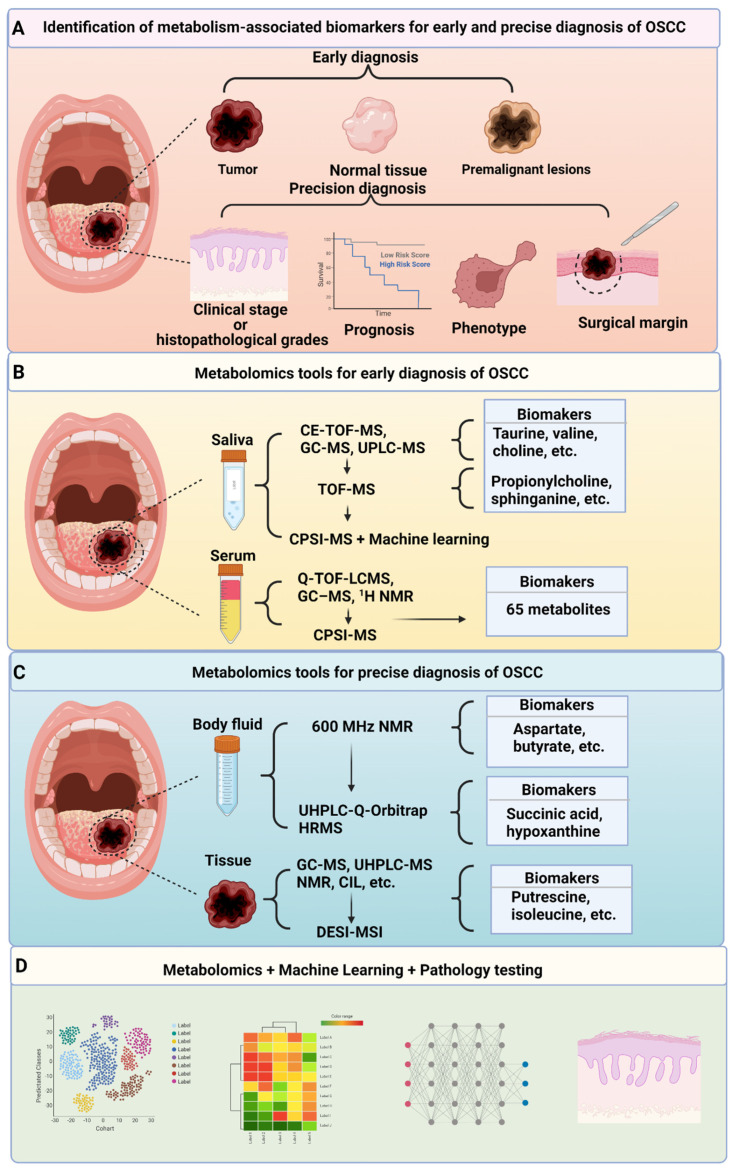
Clinical application of metabolomics to OSCC diagnosis. (**A**). Screening for metabolism-associated biomarkers to assist in early and precise diagnosis of OSCC; (**B**,**C**). With the advancement of metabolomics tools, some metabolism-associated biomarkers for early and precise diagnosis of OSCC have been screened by testing saliva, serum, body fluid and tissue samples; (**D**). Constructing a diagnostic model combined metabolomics with machine learning and pathology testing is valuable. Figure created with biorender.com.

From the perspective of clinical needs, the use of molecular markers to identify signs of progression of oral premalignant lesions to OSCC in a timely manner, or to accurately screen OSCC patients at the time of initial diagnosis, can effectively halt OSCC progression. For patients with a clear diagnosis of OSCC, identifying small tumour foci that remain at the surgical margins, or predicting therapy responses, will improve the quality of life and prolong survival (Figure 2). The current screening tools, which are primarily based on imaging to discriminate OSCC, have shortcomings. High false-positive and false-negative rates limit their clinical application. In addition, the lack of objective evaluation data also limits their development. OSCC patients undergo significant metabolic reprogramming. The identification of differential metabolites through easily accessible fluids with non-invasive or minimally invasive tools will provide a new avenue for the evaluation of OSCC as an adjunctive diagnostic technique. Although great progress has been made in metabolite-targeted OSCC diagnosis, there is still a long way to go. Based on this review, we suggest that researchers (1) develop standardised sample collection procedures; (2) focus on OSCC patient-specific metabolites, especially differential metabolites in OSCC and pre-cancerous lesions; (3) explore the association between metabolite signatures and clinical characteristics or prognosis to develop metabolic grading criteria; (4) develop economical, rapid and technologically insensitive metabolomics tools; and (5) use a combination of multidisciplinary tools, such as AI, optical coherence tomography and immune cell infiltration analysis.

## 5. Conclusions

Since OSCC induces significant metabolic reprogramming, screening for differential metabolites may assist in the diagnosis of this disease. Progress has been made in metabolomic diagnosis of OSCC; however, many problems remain unsolved. Further validation and optimisation of known metabolic diagnostic markers are necessary, and hence there is still a lot to be done.

## Figures and Tables

**Table 1:** Metabolites in OSCC.

Comparison	Upregulated Metabolites	Downregulated Metabolites	Metabolite Analysis Technique	References
OSCC patients versus healthy individuals	Choline, betaine, pipecolinic acid	l-carnitine	UPLC-MS	[98]
	Choline, p-hydroxyphenylacetic acid, 2-hydroxy-4-methylvaleric acid, valine, 3-phenyllactic acid, leucine, hexanoic acid, octanoic acid, terephthalic acid, γ-butyrobetaine, 3-(4-hydroxyphenyl) propionic acid, isoleucine, tryptophan, 3-phenylpropionic acid, 2-hydroxyvaleric acid, butyric acid, cadaverine, 2-oxoisovaleric acid, N6,N6,N6-trimethyllysine, taurine, glycolic acid, 3-hydroxybutyric acid, heptanoic acid, alanine	Urea	Capillary electrophoresis-MS (CE-MS)	[100]
	Lactic acid, hydroxyphenyllactic acid, *N*-nonanoylglycine, 5-hydroxymethyluracil, succinic acid, ornithine, hexanoylcarnitine, propionylcholine, carnitine	4-Hydroxy-L-glutamic acid, acetylphenylalanine, sphinganine, phytosphingosine, S-carboxymethyl-L-cysteine	Reversed phase liquid chromatography and hydrophilic interaction chromatography	[102]
	Putrescine, cadaverine, thymidine, adenosine, 5-aminopentoate	Hippuric acid, phosphocholine, glucose, serine, adrenic acid	Conductive polymer spray ionization mass spectrometry (CPSI-MS) and desorption electrospray ionization MS imaging (DESI-MSI)	[103]
	1-methylhistidine, pseudouridine, inositol 1,3,4-triphosphate, d-glycerate-2-phosphate, 4-nitroquinoline-1-oxide, 2-oxoarginine, norcocaine nitroxide, sphinganine-1-phosphate	l-homocysteic acid, ubiquinone, neuraminic acid, estradiol valerate	Q-TOF-liquid chromatography-MS (Q-TOF-LC-MS)	[104]
	Glutamate, aspartic acid, proline		GC-MS and ultrahigh-performance liquid chromatography-tandem MS (UHPLC-MS/MS)	[105]
	Propionate, acetone, acetate, choline	Valine, threonine, Gln, creatinine	^1^H NMR	[106]
	Malic acid, maltose, methionine, inosine		GC-MS	[107]
	Lactic acid, eicosanoic acid	Valine, γ-aminobutyric acid	Ultraperformance liquid chromatography and Q-TOF-MS	[108]
	Estradiol-17-β-3-sulfate, L-carnitine, 5-methylthioadenosine, 8-hydroxyadenine, 2-methylcitric acid, putrescine, estrone-3-sulfate		Q-TOF-LC-MS	[109]
	PC, DG, sphingosine-1-phosphate, oleamide	LysoPC (18:3), lysoPC (20:4), lysoPE (20:3/0:0), lysoSM (d18:1), erythritol, nonanovlcamitine	CPSI-MS	[110]
	TC, HDL, LDL		Automated biochemistry analyser	[111]
OSCC patients versus premalignant lesions individuals	Putrescine, cadaverine, thymidine, adenosine, 5-aminopentoate	Hippuric acid, phosphocholine, glucose, serine, adrenic acid,	CPSI-MS and DESI-MSI	[103]
	lactic acid	valine, phenylalanine	UPLC	[108]
	5,6-Dihydrouridine, 4-hydroxypenbutolol glucuronide, 8-hydroxyadenine, putrescine		Q-TOF-LC-MS	[109]
	Trimethylamine *N*-oxide, putrescine, creatinine, 5-aminovalerate, pipecolate, *N*-acetylputrescine, γ-butyrobetaine, indole-3-acetate, *N*_1_-acetylspermine, 2’-deoxyinosine, ethanolamine phosphate, *N*-acetylglucosamine	*N*-acetylhistidine, o-acetylcarnitine	CE-MS	[112]
	Acetone, acetate, choline	Valine, Gln, creatinine	^1^H NMR	[106]
OSCC tissue versus adjacent normal tissue	Lactate	Glucose	Metabolic bioluminescence imaging	[113]
	Aspartic, asparagin		GC-MS and UHPLC-MS/MS	[114]
	Carnitine,	Alanine, pyruvate	NMR	[115]
	putrescine, glycyl-leucine, phenylalanine,		Chemical isotope labeling	[116]
	stearic acid (18:0), sPLA2	Oleic acid (18:1n-9), linoleic acid (18:2n-6)	Gas liquid chromatograpy	[117]
OSCC tissue versus margin-2 (0.5–1 cm)	Aspartic acid, glutamate, proline, valine		GC-MS and UHPLC-MS/MS	[114]
margin-1 (0–0.5 cm) versus margin-2	Proline, alanine, serine, aspartic acid, glutamate, Gln, ornithine, histidine, asparagine		GC-MS and UHPLC-MS/MS	[114]
Extranodal extension (ENE)-positive versus ENE-negative	Aspartate, butyrate, carnitine, glutamate, glutathione, glycine, glycolate, guanosine, sucrose	Alanine, choline, glucose, isoleucine, lactate, leucine, myo-inositol, *O*-acetylcholine, oxypurinol, phenylalanine, pyruvate, succinate, tyrosine, valine, xanthine	600-MHz NMR	[115]
